# Revolutionizing Smiles: Advancing Orthodontics Through Digital Innovation

**DOI:** 10.7759/cureus.64086

**Published:** 2024-07-08

**Authors:** Ruchika Pandey, Ranjit Kamble, Harikishan Kanani

**Affiliations:** 1 Orthodontics and Dentofacial Orthopedics, Sharad Pawar Dental College and Hospital, Datta Meghe Institute of Higher Education & Research, Wardha, IND; 2 Pediatric Dentistry, Sharad Pawar Dental College and Hospital, Datta Meghe Institute of Higher Education & Research, Wardha, IND

**Keywords:** digitization, digitalization, digital dentistry, cad cam, digital orthodontics

## Abstract

Orthodontics is undergoing a digital revolution, transforming traditional techniques with modern technology. This evolution is driven by the need for precise diagnosis and treatment planning. Digital platforms, including digital radiography and cone beam computed tomography (CBCT), are replacing conventional methods, enhancing documentation, analysis, and appliance production. Three-dimensional imaging enables customized treatment plans and appliance design using computer-aided design and computer-aided manufacture (CAD/CAM). Integration of digital models and software facilitates treatment simulation and patient communication. Digital videography enhances diagnostic capabilities. Embracing digital processes is essential for improved patient care and practice efficiency in orthodontics. This review article on digital orthodontics aims to provide a comprehensive overview and critical analysis of the current advancements, technologies, applications, benefits, and challenges in the field of orthodontics utilizing digital tools and technologies.

## Introduction and background

Orthodontics is a specialty that gets digitalized with the rest of the globe. Computer literacy has advanced significantly, and the use of technology in orthodontics has increased dramatically. The limitations and disadvantages of traditional orthodontic office management techniques were numerous. The requirement of the future and the need of the hour is to own and operate a digital orthodontic office [1]. One of the most complicated areas of dentistry is orthodontics and dentofacial orthopedics, which necessitates the meticulous evaluation of a lot of data to arrive at an accurate diagnosis and develop a treatment plan. With the introduction of modern technologies, it has undergone a tremendous metamorphosis. The field of digital orthodontics comprises many methods and instruments that utilize digital platforms to improve patient outcomes, treatment planning, and diagnosis. Significant effects of the recent digital revolution have also been seen in orthodontics. Conventional physical imaging techniques have been supplanted by digital radiography and digital photos, and concurrent cone beam computed tomography (CBCT) is being used more and more [1].

Every area of orthodontics has been impacted by the present surge in digital processes in orthodontic practices, which include changes to documentation, study casts, malocclusion analysis, smile design, treatment planning, and orthodontic appliance production. Three-dimensional (3D) imaging of the face, skeleton, and dentition makes it possible to plan treatments in three dimensions and to customize orthodontic appliances using computer-aided design (CAD) and computer-aided manufacture (CAM). Integrating digital models, CBCT, and 3D facial imaging with software enables the simulation of treatment plans and fosters effective patient communication. Advances in digital videography have also made it possible for clinicians to simultaneously record patients' oral and throat function, speech, and smiles [2].

## Review

Digitalization in diagnosis

Orthodontic patients have precise, high-quality, and erectly posed 2D extraoral and intraoral pictures. However, they are impacted by several factors, such as the distance and angle at which the photo is taken, and do not have adequate diagnostic information. The conventional 3D topography of a patient's face surface anatomy has recently been made possible by facial scanners. This, along with a digital model and CBCT image, allows for creating a full 3D (dimensional) virtual patient [3].

A thorough evaluation of the patient's skeletal and dental structures made by digital imaging also makes it easier to determine the patient's orthodontic demands. Orthodontists are highly skilled in accurately assessing skeletal irregularities, occlusal connections, tooth alignment, and any dental pathology or anomalies that may be present [3].

Intraoral Scanners

**Figure 1:**
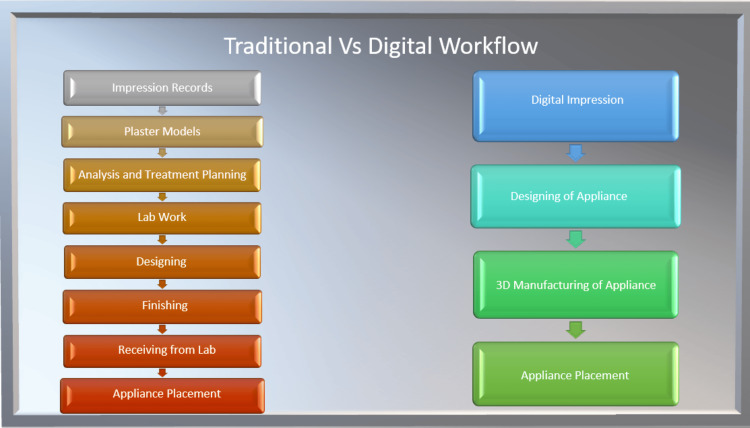
Traditional vs. digital workflow Image credits: Ruchika Pandey

Intraoral scanners have revolutionized orthodontic practices by replacing traditional impression materials with precise 3D imaging of patients' dentition. These scanners generate realistic digital models instantly, saving time, space, and costs associated with conventional methods (Figure 1). They also serve as potent marketing tools, enhancing communication with patients. Popular options include the TRIOS Classic (3Shape, Copenhagen, Denmark), CEREC Omnicam (Dentsply Sirona, Charlotte, North Carolina), and iTero Element (Align Technology, San Jose, California). Extraoral scanners offer a comfortable alternative, particularly for patients with a strong gag reflex, capturing detailed facial morphology without intraoral discomfort [4].

Laser Scanning

A non-invasive technique for capturing soft tissue and facial morphology, laser scanning is useful for evaluating facial symmetry in patients with cleft lip and palate, as well as teenagers. It has demonstrated a low error rate in precisely measuring changes in soft tissues after therapy. Its disadvantage, though, is that it takes a long time to scan, which makes it difficult for pediatric situations. However, a recent study found that it can still be appropriate for preschoolers with the proper planning [5].

Magnetic Resonance Imaging (MRI)

When evaluating craniofacial anomalies, MRI is a useful diagnostic technique that provides thorough information on both soft and hard structures, especially in the temporomandibular joint (TMJ). It is commonly used for upper airway studies and 3D imaging of TMJ anatomy. In particular, MRI is useful for assessing velopharyngeal function because it yields precise information on airway mobility and space, especially in patients with cleft palates. Recent studies have shown the usefulness of MRI in numerous orthodontic applications and have compared its effectiveness to conventional 3D imaging techniques such as CBCT and CT despite early reservations regarding its applicability and cost [5].

CBCT Scans

CBCT scans can be used when a thorough assessment of the skeletal and oral structures is necessary. Orthodontists can evaluate tooth roots, bone morphology, and any pathology that could affect treatment planning with the help of computed tomography (CBCT), which produces 3D images of the teeth, jaws, and surrounding tissues. The basic 3D reconstruction's multiplanar reformatting (MPR) enables investigations of any chosen plane in 2D or 3D views [6]. To analyze the spatial disposition (angle, length, and diameter) of mini-implants, infra-zygomatic crest screws, and orthodontic components, raw CBCT axial images are rendered into 3D reconstructed models. It is necessary to receive the raw CT or CBCT data in the DICOM® (Digital Imaging and Communication in Medicine) extension [7]. Currently, a variety of software applications can be used to register (superimpose) patient CBCT scans and scanned pictures of patient dental casts. The end product is a 3D computerized composite image of the skull that includes the occlusion. This image serves as the foundation for virtual planning in orthognathic surgery. Surgical guides, splints, personalized plates, and prostheses can be used to transmit the virtual plan to the operation room through subtractive or additive direct digital manufacturing procedures [8].

Digital Models

Transitioning to 3D virtual or digital orthodontic models has many benefits, including improved precision and easier practitioner communication. These virtual models provide accurate measurements, ease interdisciplinary treatment planning, and lessen the wear and breakage problems that come with plaster models. The ability to conduct thorough space analysis and generate indirect bracket bonding setups provides orthodontic practice with even more efficiency. Additionally, the ease with which these virtual models may be shared via email encourages cooperation between various dental specialties, which eventually improves patient care. Furthermore, patients' comprehension of the course of therapy and its results can be substantially enhanced using virtual treatment objective (VTO) communication. These virtual models provide unmatched flexibility, whether acquired directly from intraoral scanning or indirectly through plaster models. The software's capacity to work with models in several spatial planes facilitates in-depth inspection and analysis, which improves treatment planning and communication to a greater extent [1,9].

Digitalization in treatment planning

In orthodontics, treatment planning software improves treatment efficiency and results by gathering and evaluating patient data from multiple sources, including radiographs, digital impressions, and clinical assessments. It consists of virtual model analysis for tracking advancement, cephalometric analysis for assessing skeletal relationships, and digital imaging techniques for modeling therapy effects [10]. The program interfaces with practice management systems to simplify workflow and enables the customization of treatment regimens based on unique patient features. It also offers educational tools that can be used effectively and promotes multidisciplinary collaboration. In orthodontic practice, treatment planning software enhances professional judgment, communication, and patient satisfaction overall.

Three major kinds of orthodontic software are available, each meeting a distinct need: programs for creating 3D digital models, modeling orthodontic treatments, and conducting analysis and treatment programming. Depending on the situation, an orthodontic digital workflow can call for utilizing all three technologies at once, or it might just call for employing one or the other [11].

Modern dental scanners incorporate machine learning and artificial intelligence to precisely discern textures and colors, which expedites file preparation. With its ability to provide visualization tools for patient communication, orthodontic software for treatment simulation is a logical progression in workflow. These methods require little input data, whether modeling clear aligner or bracket treatments [12].

A wide range of features are available in orthodontic practice software and digital treatment planning systems, such as digital imaging, tools for case presentation and treatment planning, morphing capabilities for treatment outcome visualization, intraoral and extraoral image capture, and cephalometric analysis. Cephalometric analysis has been transformed by artificial intelligence (AI), which has increased efficiency and accuracy over manual techniques. AI applications include skeletal age determination, TMJ evaluation, extraction decision-making, planning orthognathic surgery, and treatment result prediction. Practitioners can assess various treatment approaches, such as replacement versus extraction in cases of missing teeth or extraction versus non-extraction treatment choices. They also make applications for oral and maxillofacial surgery easier [13].

Digitalization in appliance fabrication

In orthodontics, CAD is revolutionary. The field of orthodontics has dramatically changed with the adoption of CAD software and the digitization of the oral cavity. Through this procedure, dentists can digitally design and modify appliances before using 3D printers to manufacture them. Because of scanners, computers, and printers, conventional orthodontic labs are evolving into digital ones with less equipment and a cleaner atmosphere.

This technology even enables in-office production, where everything from planning to printing may occur in the orthodontist's office. The central component of this digital lab is the computer, which manages all design and printing functions. With the precise customization made possible by this technology, every patient's particular needs can be fully met. Orthodontists can create a wide range of appliances, including the newest ones: digital models, customized brackets with torque specific to the patient, machine-milled indirect bonding trays, robotically bent archwires, occlusal splints, orthodontic bands, and thermoformed and printed aligners. These days, the usage of aligners in orthodontic treatment has significantly increased since the advent of CAD/CAM technology. Most appliances can be printed using VAT technology, or for metal appliances, powder bed fusion printers can be contracted out to handle the printing [14].

The use of 3D printers and intraoral scanners eliminates the need for dental impressions and processes such as gypsum casting in various orthodontic workflows, resulting in a fully digitalized workflow. Some innovative brands in 3D printing for orthodontics include Stratasys (Eden Prairie, Minnesota), which is a leading maker of 3D printers and materials used in a range of industries, including orthodontics. Stratasys provides a variety of 3D printers that can be used to make orthodontic models, aligners, and surgical guides. EnvisionTEC (Gladbeck, Germany) is a company that focuses on 3D printing for medical and dental purposes. EnvisionTEC provides a variety of 3D printers that are ideal for orthodontics. Carbon3D (Redwood City, California) is a company that specializes in high-speed 3D printing technology. Carbon3D (Digital Light Synthesis) printers can produce high-quality orthodontic models and aligners. Desktop Metal (Burlington, Massachusetts) is a 3D printing firm focusing on metal. Desktop Metal's (Studio System) technology may be used to make custom orthodontic brackets or other metal components [15].

Dentists can also simulate treatment outcomes with CAD software, which provides them with a clear understanding of what to expect prior to beginning treatment. This increases accuracy and facilitates a better understanding of the procedure for the patients. CAD speeds up the production process, saving patients' wait periods. It also saves time and resources by enabling simple adjustments when necessary [16].

The manufacturing of orthodontic appliances has changed due to CAM, mainly due to its accurate and effective milling procedure. CAD software is used for digital design at the outset, which enables precise customization based on each patient's requirements [17].

After the design is complete, CAM software transforms the digital model into instructions that the milling machine can understand, preparing it for milling. The appliance is carved out of a metal or ceramic block during the milling process. Because of the extreme precision of this procedure, appliances fit and operate flawlessly. Additionally, CAM makes careful quality control during manufacture possible, guaranteeing that every appliance fulfills precise specifications for the best possible treatment results. Patient wait times are decreased, and CAM makes quicker procedures possible, which is more efficient and faster than previous methods [18].

Aligners

Today's widely utilized clear aligners are usually made via a procedure that starts with virtual modeling and finishes with 3D printing. This makes use of CAM technology and either additive or subtractive manufacturing methods. Currently, the predominant technique is 3D printing, which has benefits such as increased accuracy, lower costs, and possibly less environmental influence. A noteworthy development is the ability to directly 3D print from digital designs, which enables customized thickness and spatial control of aligners along the arch. 3D printing can be done using various materials, while photopolymerization from clear resins shows promise. All things considered, this procedure improves precision, expedites output, and provides long-term advantages for orthodontic care [19].

Clear aligners have revolutionized orthodontics by offering a discreet alternative to conventional fixed appliances. Recent advancements have significantly enhanced their effectiveness, comfort, and customization, making them a compelling choice for patients seeking a cosmetically pleasing approach. One key area of progress is the development of innovative materials that improve both comfort and treatment efficiency. For instance, Ghost PU Aligner (Pū) Sheets represent a major leap forward. These sheets, crafted from a highly durable polyurethane, provide an ideal balance of flexibility and strength. This allows them to exert consistent and controlled force on teeth, leading to faster and more predictable results. Additionally, visual tests confirm excellent color stability, even after exposure to common staining agents such as coffee, wine, and nicotine [19].

Invisalign appliances, particularly, have undergone a remarkable transformation since their inception. The current eighth-generation aligners represent a significant evolution from their first-generation counterparts. The most noteworthy advancement lies in material advancements, such as the introduction of SmartTrack, a multi-layered material combining aromatic thermoplastic polyurethane and co-polyester. Furthermore, the latest generation boasts "SmartForce" features, which allow for incorporating various attachments and auxiliaries based on simulations generated by clinical software. Software enhancements also play a crucial role. These advancements include the ability to integrate CBCT scans, utilize separate software for extraction and mixed dentition cases, and facilitate lower jaw advancement for correcting Skeletal Class II malocclusions [20].

Lingual Orthodontics

Lingual orthodontics has been transformed by CAD/CAM technology, especially in the milling part of appliance construction. Digital impressions of the patient's teeth are the first step, and CAD software is used to convert them into exact 3D models. With the aid of these computerized models, orthodontists may create lingual brackets and wires that are specifically suited to each patient's anatomy and course of treatment. Once the design is finished, the milling machine is prepared for milling using CAM software to convert the digital model into instructions. Using a milling technique, the brackets and wires are carved out of a block of material, such as titanium, stainless steel, or gold. With this process, the best fit and effectiveness are ensured for the duration of treatment, and the highest level of accuracy and precision in creating lingual orthodontic equipment is achieved [21].

Archwire Fabrication

One significant advancement in orthodontic therapy is using robotics to bend and manufacture archwires. In the past, archwires had to be manually bent, which is a tedious and uncertain process. However, the application of robotic technology has revolutionized this significant aspect of orthodontic therapy. There are limits to hand bending; robotic alternatives offer accuracy and automation. With the use of intricate algorithms and programming, these robots can precisely bend archwires in compliance with the individual treatment plans for each patient. Ensuring wire size, curvature, and force delivery homogeneity improves treatment outcomes. Moreover, robotic archwire bending increases treatment efficiency by reducing the amount of time and manpower required during production. In addition to improving patient comfort, orthodontists can concentrate more on treatment planning [22,23].

In 2019, LightForce Orthodontics (LightForce, Burlington, Massachusetts) revolutionized braces with the first 3D-printed system. Unlike traditional one-size-fits-all brackets, LightForce uses LightPlan software to create fully customized brackets for each tooth, including molars, using 3D-printed polycrystalline alumina. This allows for a more precise fit and treatment plan, potentially reducing treatment time and appointments. LightForce also offers customizable features such as slot size, color, and bite turbos for increased comfort and control. By leveraging 3D printing and focusing on customization, LightForce provides a more efficient and potentially more comfortable path to a beautiful smile [24].

Surgical Splint

Technological developments have completely changed the production of surgical splints and preoperative planning. By improving the assessment of complicated abnormalities, preoperative planning quality can be improved by switching from 2D to 3D imaging. Surgeons can engage with 3D pictures using various software programs, which simulate surgery and anticipate postoperative outcomes. Using CAD/CAM technology, surgical splints ensure optimal results by preventing errors encountered in older approaches. Furthermore, CBCT technology helps assess the results of combination orthodontic and surgical therapies. These polymethyl methacrylate splints provide precise guidance during surgery to provide the best possible outcome [25].

Retainers

Following orthodontic treatment, retention is essential to preventing tooth recurrence. There are several approaches available, each with advantages and disadvantages. Studies indicate that the use of digital techniques yields better results than normal procedures when it comes to creating lingual retainers. While CAD/CAM retainers have smoother surfaces and lessen plaque buildup and inflammation, stainless-steel retainers are known to enhance plaque accumulation and gingival inflammation. Because fixed lingual retainers require less patient cooperation than traditional removable ones, they have supplanted them; an alternative option is invisible retainers, providing reduced breakage, a perfect fit, and an optimum transfer to the intended place. Retainers that are thinner and thicker have greater bending resistance than ordinary ones. Because they are simple, quick, affordable, and repeatable, 3D-printed retainers are a promising replacement for metal ones [26].

Other Appliances and Auxiliaries

An important development in orthodontic technology is the use of digital fixed appliances, which enable accurate and personalized treatment plans. Fully digital lingual arches, trans-palatal arches, sectional space holders, and customized anchorage structures are just a few examples. Lingual tongue spurs can be used for tongue-thrusting habits, and Herbst and Forsus appliances can be used to treat faulty positioned mandibles. Furthermore, Class III appliances can also be digitally customized, offering design freedom in accordance with clinical requirements. The development of digital bands lessens patient discomfort by eliminating the necessity for separator elastics because they are perfectly tailored to fit tooth surfaces. Digital bands reduce the chance of gingival inflammation because they snugly conform to the edges of teeth without stretching between contact areas like traditional bands do [27].

The Digital Titanium Herbst (DTi Herbst, Sheboygan, Wisconsin) appliance is a leading-edge orthodontic device that addresses jaw discrepancies using innovative materials and digital design. Unlike previous Herbst equipment, which is frequently composed of metal, DTi Herbst uses titanium, a biocompatible, robust, and perhaps more pleasant material. The digital aspect is addressed through the use of CAD and CAM processes. This enables each DTi Herbst device to be custom-designed for a precise fit depending on the patient's specific requirements. This digital approach has the potential to not only enhance comfort but also lead to shorter treatment times [28].

By simplifying the design and manufacturing process, digital removable appliances, such as vacuformed retainers, are revolutionizing orthodontic therapy. 3D printing advancements hope to print high-quality plastic directly, eliminating the need for thermoforming entirely. Precise digital modeling is beneficial for functional equipment, which is necessary for bite correction. Technicians guarantee precise appliance construction using virtual design and bite registration in an ideal mandibular position. It is possible to incorporate extra elements, such as labial arches or Adams clasps, into the design without notice. Michigan splints, which are essential for treating joint diseases, can be customized through digital design. Digitally designed tongue educators for lingual training conform to the palate and improve treatment results [27]. A table summarizing a few research and case reports is provided below to support the literature evidence [Table 1]. Further investigation is necessary to reach a definitive judgment.

**Table 1:** Few research and case reports to support the literature

Author	Year	Research/Article
Nucera et al.	2023 [29]	Presented two cases where Digital Bonded Twin Block was used to correct skeletal class II malocclusion in children with mixed dentition. It is a new approach to orthodontic treatment for skeletal malocclusion in children. It offers the potential benefit of removing reliance on patient cooperation for wear time.
Guidice et al.	2020 [30]	Presented a case report on how orthodontists can plan maxillary skeletal expander placement based on a patient's specific anatomy using CBCT scans. The digital plan helps ensure mini-screws (tiny screws used for anchorage) are placed in optimal positions for bone thickness and biomechanical effects.
Fiorillo et al.	2023 [31]	Presented a study on a new technique for bonding orthodontic brackets using computer-aided design and manufacturing (CAD/CAM). It utilizes a customized 3D-printed transfer tray and a special adhesive for accurate bracket placement. The research found this technique generally accurate, although with slightly higher error rates for brackets on back teeth.
ElShebiny et al.	2024 [32]	Did a study to investigate the use of 3D printing technology by orthodontists in North America. According to the study, around 75% of orthodontists in North America use 3D printing technology in some way. The primary reasons orthodontists use 3D printing are self-interest and research. Orthodontists mainly use 3D printers to create plastic retainers from printed models.
Pouliezou et al.	2024 [33]	Presented an article discussing the growing importance of digital models in orthodontics. These combine facial scans, jaw structures (craniofacial), and teeth information from a patient to create a virtual representation. Different digital scans (CBCT, dental scans, facial scans) are merged using specific software.
Awad et al.	2022 [34]	Did a study to evaluate how much facial soft tissue changes after orthognathic surgery. Realistic expectations can improve patient satisfaction. It can be concluded that the VSP system IPS CaseDesigner predicts the patient’s operative soft tissue accurately.

Challenges and limitations

Despite the fact that digitization has completely changed orthodontic practices and has many advantages, there are still some significant obstacles to overcome. First, the substantial upfront costs associated with software, hardware, and training provide a financial challenge, especially for smaller clinics. Furthermore, orthodontists and staff may need to invest time and money due to the steep learning curve involved in mastering digital tools and software. Even with great accuracy promised, mistakes and variances in digital impressions, CAD/CAM designs, and 3D printing can still occur, necessitating careful calibration and cross-checking. Incorporating digital technology into current workflows may lead to inefficiencies and the need to modify procedures.

Considering the difficulties brought on by regulatory requirements and cyber dangers, it is essential to protect sensitive patient data in digital records. The standardization and platform compatibility issues that impede collaboration and data transfer necessitate a planned approach, such as training practitioners on cybersecurity measures and informing them about breaches, access controls, data encryption, etc. Developing a culture of effective teamwork via education and communication is crucial given the varying degrees of digital technology adoption among patients. It is essential for practitioners to give preference to patient education. Adherence to changing standards is crucial in order to fulfill legal responsibilities, thus adding to the difficulty of practice management. For digital hardware and software to perform at their best, regular maintenance and professional assistance are also essential. With these challenges, if digital orthodontics is developed with caution and flexibility, it has the potential to greatly enhance patient care and productivity with innovations and advancements.

## Conclusions

Digital orthodontics transforms the discipline by providing professionals with innovative instruments and methods to enhance patient care. Intraoral scanners, 3D imaging, and CAD are examples of digital technology that simplify patient-orthodontist communication while simplifying diagnostic and treatment planning. Through the adoption of this technology and continued education and training, practitioners may enhance patient outcomes and deliver high-quality care. Data security has to be prioritized in order to protect patient privacy. All things considered, digital orthodontics presents a wonderful opportunity for practitioners to raise their standards and improve treatment efficacy.
